# Cre8 Drug Eluting Stent Performance in Daily Cardiology Practice

**DOI:** 10.31083/j.rcm2402053

**Published:** 2023-02-06

**Authors:** Umit Yasar Sinan, Ebru Serin, Bengisu Keskin-Meric, Alev Arat-Ozkan

**Affiliations:** ^1^Department of Cardiology, İstanbul University Cardiology Institute, 34096 Istanbul, Turkey

**Keywords:** Cre8, drug eluting stent, performance, target lesion failure, device-oriented primary end-point

## Abstract

**Background:**

In patients undergoing percutaneous coronary intervention (PCI), drug eluting stents (DES) are currently the standard of care. Stent design and alloy composition, biocompatibility of the drug-eluting polymer coating, the antiproliferative agent properties and release are the three main characteristics that affects DES performance. Cre8 (Alvimedica, Istanbul, Turkey) is a polymer-free amphilimus-eluting stents (PF-AES). In this study, we aimed to investigate the clinical efficacy and safety of Cre8 DES in daily cardiology practice.

**Methods:**

Patients presenting with chronic coronary syndrome (CCS) or acute coronary syndrome (ACS) including unstable angina pectoris (USAP), myocardial infarction with and without ST-segment elevation and treated with PCI using Cre8 DES between December 2015 and 2016 were retrospectively analyzed in this study.

**Results:**

Between December 2015 and 2016, 808 lesions of 664 patients treated with Cre8 DES in a single center were included in this retrospective analysis. The mean age of study group was 60 years (between 33 and 93 years) and were predominantly consisting of male patients (79.4%). The median follow-up duration was 487 days (min: 30 days, max: 919 days) and two-thirds of all patients presented with ACS. The culprit lesion was on left anterior descending artery (LAD) (40.5%) and right coronary artery (RCA) (25.9%) in most of the patients. The procedural success rate was 97.3%. Most of the lesions were type B1 (40.6%) according to American College of Cardiology/American Heart Association (ACC/AHA) coronary lesion classification. The device oriented primary end-point defined as target lesion failure (TLF) occurred in 52 (6.4%) of 808 lesions. The primary safety end-point was cardiac death in 20 patients (3.0%) and target vessel myocardial infarction in 2 patients (0.3%). Target vessel revascularization (TVR) occurred in 29 patients (4.4%) as primary safety endpoint. Multivariable logistic regression analysis revealed diabetes mellitus and ejection fraction as the predictors of mortality and device oriented primary end-point.

**Conclusions:**

This trial revealed clinical efficacy and safety of Cre8 stents in real world practice. Device oriented primary end points were similar with previous studies which are randomized, open label in nature and showed the efficacy and safety of Cre8 stent towards latest generation DES.

## 1. Introduction

Drug eluting stents (DES) are the current standard of care in patients undergoing percutaneous coronary intervention (PCI). The restenosis rate of DES is lower than bare metal stents (BMS), as antiproliferative agents are released by DES platforms [1]. Stent design and alloy composition, biocompatibility of the drug-eluting polymer coating, the antiproliferative agent properties and release are the three main characteristics that affect DES performance.

Increased risk of late stent thrombosis is major problem for DES as permanent polymer content causes incomplete stent strut endothelialization due to impaired arterial healing especially in patients with comorbidities and complex lesions [2]. Contrarily, late restenosis rate of polymer-free amphilimus-eluting stents (PF-AES) is less than permanent polymer paclitaxel-eluting stent [3]. Cre8 (Alvimedica, Istanbul, Turkey), a PF-AES, was non-inferior to latest generation permanent-polymer zotarolimus-eluting stents (PP-ZES) regarding target lesion failure at 12 months in Randomized All-Comers Evaluation of a Permanent Polymer Zotarolimus-Eluting Stent Versus a Polymer-Free Amphilimus-Eluting Stent: a Multicenter, Noninferiority Trial (ReCre8) [4].

The use of Cre8 in acute coronary syndrome (ACS) patients is poorly investigated, especially in the myocardial infarction with ST segment elevation (STEMI) scenario. In this study, we aimed to investigate the clinical efficacy and safety of Cre8 DES in daily cardiology practice.

## 2. Material and Methods

This is a retrospective, observational, single center study. Patients presenting with chronic coronary syndrome (CCS) or ACS including unstable angina pectoris (USAP), myocardial infarction with and without ST-segment elevation and were treated with Cre8 DES between December 2015 and 2016 at Istanbul University Institute of Cardiology were retrospectively analyzed in this study. All lesion treated with Cre8 and classified as American College of Cardiology/American Heart Association (ACC/AHA) class A, B1, B2 and C according to lesion complexity were included in the final analysis. There was no restriction for lesion types, lengths, or number of treated lesions. The duration of dual antiplatelet therapy (DAPT) was in accordance with current guideline.

Procedural success was defined as less than a <50% residual stenosis with antegrade thrombosis in myocardial infarction (TIMI) flow grade 3 at the end of the procedure. While the primary efficacy end-point was target vessel revascularization at follow-up, for safety the end-point was target-lesion failure (TLF) was defined as a cardiac death and target vessel myocardial infarction. Death was accepted as cardiac when there was a clear cardiac evident or related to PCI, unwitnessed death, or death due to unknown aetiologies. For the term of myocardial infarction (MI), fourth universal definition of MI was used [5]. Target lesion revascularization (TLR) was defined as any repeat PCI or coronary bypass surgery due to >50% stenosis within a 5-mm border adjacent to the study stent. Target-vessel revascularization (TVR) was defined as a repeat PCI or bypass surgery in the territory of the coronary artery which includes target lesion. Revascularization was the treatment of choice if any of the target lesion or vessel showed ≥50% stenosis in the presence of objective evidence of ischemia from noninvasive or invasive testing, or symptoms. Unplanned revascularization was defined as any repeat revascularization of lesions that were not detected during the index coronary angiogram and demanded treatment by PCI. Patients characteristics, comorbidities and demographics were collected from medical records. All coronary angiographic images were evaluated by two experienced invasive cardiologists to decide lesion characteristics and periprocedural complications. The primary outcome was investigated from hospital records, outpatient visits and phone calls.

## 3. Statistical Analysis

For statistical analysis, Number Cruncher Statistical System (NCSS) 2007 (Kaysville, UT, USA) was used. Kolmogorov Smirnov test is the preferred method to evaluate the distribution of variables. The variables were expressed as mean ± standard deviation or median (interquartile range) according to distribution characteristics. Categorical variables were compared with a Chi-square test or Fisher’s exact test. The Mann-Whitney* U* test or unpaired *t*-test, as appropriate, was used to evaluate the quantitative data. Logistic regression analysis was used to determine the effect of independent categorical and quantitative variables on dependent categorical variables. Kaplan Meier method was used to compare survival. The *p *< 0.05 were considered as significant.

## 4. Results

Between December 2015 and 2016, 808 lesions of 664 patients treated with Cre8 DES in a single center were included in this retrospective analysis. The mean age of study group was 60 years (between 33 and 93 years). All patients were treated with at least one Cre8 DES. The median follow-up duration was 487 days (min: 30 days, max: 919 days). The study group predominantly consisted of male patients (79.4%) and two-thirds of all patients presented with ACS. Baseline characteristics are shown in Table 1.

**Table 1.** **Baseline characteristics**.

		Overall (n = 664)
Clinical characteristics		
	Age (years)	60.2 ± 10.7
	Male sex (n, %)	527 (79.4)
	Diabetes mellitus (n, %)	246 (37.0)
	Hypertension (n, %)	338 (50.9)
	Hypercholesterolemia (n, %)	90 (13.6)
	Coronary artery disease (n, %)	329 (49.5)
	Current smoker (n, %)	171 (25.8)
Clinical presentation (n, %)		
	Chronic coronary syndrome	279 (42.0)
	Acute coronary syndrome	385 (58.0)
	Unstable angina	101 (15.2)
	NSTEMI	119 (17.9)
	STEMI	165 (24.1)
Coronary anatomy (n, %)		
	Left main	8 (1.0)
	Left anterior descending artery	342 (42.4)
	Left circumflex artery	204 (25.2)
	Right coronary artery	220 (27.2)
	Bypass graft	34 (4.2)

Abbreviations: NSTEMI, Myocardial infarction without ST segment elevation; STEMI, Myocardial infarction with ST segment elevation.

The indication was ACS in 57.7% of patients and CCS in the rest of patients. The culprit lesion was on left anterior descending artery (LAD) (40.5%) and right coronary artery (RCA) (25.9%) in most of the patients. While direct stenting was the treatment of choice in 28.5% of lesions, most of the lesions were predilated with balloon before stenting. The mean stent diameter and length were 2.8 mm (min: 2.25 mm, max: 3.5 mm), 21.2 mm (min: 15 mm, max: 38 mm) respectively. The procedural success rate was 97.3%. Most of the lesions were type B1 (40.6%) according to ACC/AHA classification of coronary lesion. It was followed by type B2, type A, and type C 23.0%, 22.3%, and 14.1% respectively. Lesion and procedural characteristics are listed in Table 2.

**Table 2.** **Lesion and procedural characteristics**.

		Overall (808 Lesions)
Procedural characteristics		
	No of stents, per lesion	1.2 ± 0.2
	No of stents, per patients	1.4 ± 0.6
	Total stent length, mm	21.2 ± 6.1
	Stent diameter, mm	2.8 ± 0.3
	Pre-dilatation (n, %)	578 (71.5)
	Post-dilatation (n, %)	214 (26.5)
Lesion complexity (n, %)		
	ACC/AHA Class A	151 (18.9)
	ACC/AHA Class B1	330 (40.8)
	ACC/AHA Class B2	212 (26.2)
	ACC/AHA Class C	115 (14.2)
	Procedural success (n, %)	785 (97.3)

Abbreviations: ACC, American College of Cardiology; AHA, American Heart Association.

**Fig. 1.**
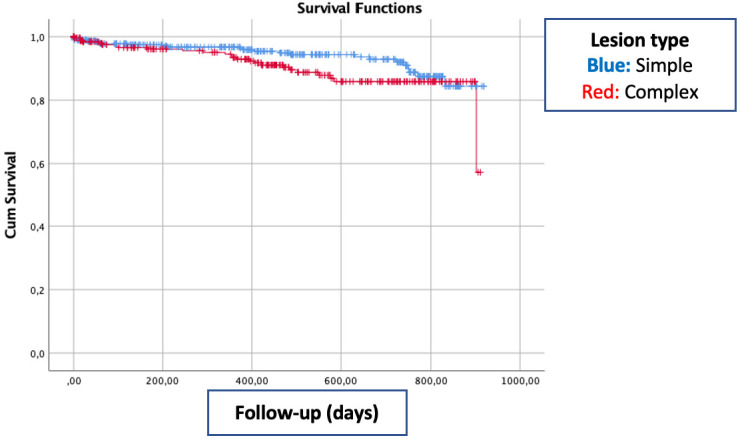
**Kaplan Meier graph of event free survival according to lesion complexity**.

The device oriented primary end-point defined as TLF occurred in 52 (6.4%) of 808 lesions. The primary safety end-point was cardiac death in 20 patients (3.0%) and target vessel myocardial infarction in 2 patients (0.3%). Target vessel revascularization (TVR) occurred in 29 patients (4.4%) as primary safety end-point. Diabetes mellitus (DM), lesion complexity (type B2 and C lesion) and ejection fraction (EF) were the predictors for the device oriented primary end-point. There was no relation between stent diameter, stent length, final diameter and event-free survival (Table 3). Multivariable logistic regression analysis revealed diabetes mellitus and ejection fraction as the predictors of both mortality and device oriented primary end-point (Table 4). Kaplan Meier Survival analysis showed no significant survival difference between patient with and without complex coronary lesion (Fig. 1).

**Table 3.** **Predictors of target lesion failure (TLF)**.

Parameter	TLF (+) (52, 6.4%)	TLF (–) (756, 93.6%)	*p* value
Age (years)	60.0 ± 10.8	60.2 ± 10.7	0.986
Sex (male) (%)	70.8	80.0	0.147
DM (%)	60.4	35.4	0.001
HT (%)	62.5	50.3	0.102
HL (%)	22.9	12.9	0.071
Smoking (%)	62.5	80.9	0.194
CAD (%)	52.1	49.5	0.731
Lesion type (B2 and C) (%)	44.3	39.1	0.017
EF (%)	49.4 ± 11.8	57±9.4	0.020
Stent diameter (mm)	2.8 ± 0.3	2.8 ± 0.3	0.788
Stent length (mm)	20.3 ± 5.4	21.2 ± 6.0	0.497
Final diameter (mm)	3.2 ± 0.4	3.2 ± 0.4	0.494

Abbreviations: CAD, Coronary artery disease; DM, Diabetes mellitus; EF, Ejection fraction; HT, Hypertension; HL, Hyperlipidemia.

**Table 4.** **The results of multivariable logistic regression analysis on device oriented primary end-point**.

Model	Variables	B	S. Error	Wald	*p*
1	Constant	–5.790	1.902	9.270	0.002**
	HT	0.299	0.556	0.290	0.590
	IHD	0.023	0.506	0.002	0.964
	Age	0.043	0.026	2.769	0.096
	HL	–0.274	0.615	0.199	0.656
	DM	–1.549	0.575	7.267	0.007**
	Sex	0.421	0.614	0.470	0.493
$R^{2}$ = 0.09, $X^{2}$ = 12.685, *p *= 0.048					
Model	Variables	B	S. Error	Wald	*p*
1	Constant	–6.088	2.617	5.411	0.02*
	Stent Diameter	1.190	0.919	1.675	0.196
	EF	–0.019	0.009	4.741	0.029*
$R^{2}$ = 0.03, $X^{2}$ = 6.037, *p* = 0.049					

Abbreviations: DM, Diabetes mellitus; EF, Ejection fraction; HT, Hypertension; HL, Hyperlipidemia; IHD, Ischemic heart disease. * *p *< 0.05, ** *p *< 0.01.

## 5. Discussion

Our study showed the clinical efficacy and safety of Cre8 stent in real-life practice. At median 16-month follow-up, the event-free survival rate was 93.6%. Although two thirds of patients presented with ACS, the mortality rate at follow-up was 3.0%. Also the procedural success rate was high (97.3%) despite that one third of patients had complex lesion (type B2 and C lesion according to ACC/AHA classification of coronary lesion classification). Diabetes mellitus, EF and lesion complexity were predictors of outcome in univariable analysis. After multivariable logistic regression analysis, DM and EF were constant predictors of outcome.

The ReCre8 study showed that Cre8 polymer-free amphilimus-eluting stents (PF-AES) (Alvimedica, Istanbul, Turkey) is clinically non-inferior to latest generation DES regarding TLF at 12 months [4]. As in our study, most of patients presented with ACS and reflected true all-comers population. Half of patients had complex lesion (type B2 and C) and the procedural success rate was 99.3% in Cre8 group. Although device oriented primary end point was lower in ReCre8 trial, (5.6% and 6.4%), our study consisted of more patients with DM (20.4% and 30.4%) and the mortality rate was similar between the two studies (2.4% and 3.0%). Also the median follow-up duration was 12-month in ReCre8 trial. Recently van Hemert *et al*. [6], presented 3-year clinical outcomes of ReCre 8 study showing PF-AES are clinically noninferior to PP-ZES regarding TLF between 1 and 3 years.

Although target lesion failure (TLF) was slightly higher than in previous studies [4, 6, 7, 8], TVR rate (4.4%) was lower in our study. Also the proportion of patients with target-vessel myocardial infarction (0.3%) was distinctly lower than that reported in previous studies (2%–6%) [4, 7, 9, 10, 11].

Despite advance in stent technology, the clinical outcomes in diabetic patients is still worse than in non-diabetics. The rate of in-stent restenosis and TLR is reaching up to 13.5% in diabetic population [12]. The amphilimus formulation consists of a mixture of sirolimus and long-chained fatty acids used in polymer free amphilimus eluting-stents and this enhances the uptake of antiproliferative agents. This property may be associated with higher anti-restenosis potency in diabetics [13]. Previous clinical studies have revealed encouraging results on PF-AES in DM [3, 14]. Patients with DM almost had two-fold increased risk for device oriented primary end point (60.4% and 35.4%) in our study. After logistic regression analysis, DM is one of the predicting factors for device oriented cardiac events.

Our study has several limitations. First of all, it was a single center retrospective study. Patients treated with Cre8 in a year period were included in this analysis. It was open label and non-randomized study. Second, there was no other group of DES as comparator. So we were not able to compare efficacy and safety of different DES.

## 6. Conclusions

This trial reveals clinical efficacy and safety of Cre8 stents in real world practice. Device oriented primary end points are similar to previous studies which are randomized, open label in nature and showed the efficacy and safety of Cre8 stent towards latest generation DES.

## Data Availability

The datasets used and/or analyzed during the current study are available from the corresponding author on reasonable request.

## References

[1] Stefanini GG, Holmes DR (2013). Drug-Eluting Coronary-Artery Stents. *New England Journal of Medicine*.

[2] Joner M, Finn AV, Farb A, Mont EK, Kolodgie FD, Ladich E (2006). Pathology of Drug-Eluting Stents in Humans: delayed healing and late thrombotic risk. *Journal of the American College of Cardiology*.

[3] Carrié D, Berland J, Verheye S, Hauptmann KE, Vrolix M, Violini R (2012). A Multicenter Randomized Trial Comparing Amphilimus- with Paclitaxel-Eluting Stents in De Novo Native Coronary Artery Lesions. *Journal of the American College of Cardiology*.

[4] Rozemeijer R, Stein M, Voskuil M, van den Bor R, Frambach P, Pereira B (2019). Randomized all-Comers Evaluation of a Permanent Polymer Zotarolimus-Eluting Stent Versus a Polymer-Free Amphilimus-Eluting Stent: a Multicenter, Noninferiority Trial (ReCre8). *Circulation*.

[5] Thygesen K, Alpert JS, Jaffe AS, Chaitman BR, Bax JJ, Morrow DA (2019). Fourth universal definition of myocardial infarction (2018). *European Heart Journal*.

[6] van Hemert ND, Voskuil M, Rozemeijer R, Stein M, Frambach P, Pereira B (2021). 3-Year Clinical Outcomes after Implantation of Permanent-Polymer Versus Polymer-Free Stent: ReCre8 Landmark Analysis. *JACC: Cardiovascular Interventions*.

[7] von Birgelen C, Sen H, Lam MK, Danse PW, Jessurun GA, Hautvast RW (2014). Third-generation zotarolimus-eluting and everolimus-eluting stents in all-comer patients requiring a percutaneous coronary intervention (DUTCH PEERS): a randomised, single-blind, multicentre, non-inferiority trial. *The Lancet*.

[8] Raungaard B, Jensen LO, Tilsted HH, Christiansen EH, Maeng M, Terkelsen CJ (2015). Zotarolimus-eluting durable-polymer-coated stent versus a biolimus-eluting biodegradable-polymer-coated stent in unselected patients undergoing percutaneous coronary intervention (SORT OUT VI): a randomised non-inferiority trial. *The Lancet*.

[9] von Birgelen C, Kok MM, van der Heijden LC, Danse PW, Schotborgh CE, Scholte M (2016). Very thin strut biodegradable polymer everolimus-eluting and sirolimus-eluting stents versus durable polymer zotarolimus-eluting stents in allcomers with coronary artery disease (BIO-RESORT): a three-arm, randomised, non-inferiority trial. *The Lancet*.

[10] de Winter RJ, Katagiri Y, Asano T, Milewski KP, Lurz P, Buszman P (2018). A sirolimus-eluting bioabsorbable polymer-coated stent (MiStent) versus an everolimus-eluting durable polymer stent (Xience) after percutaneous coronary intervention (DESSOLVE III): a randomised, single-blind, multicentre, non-inferiority, phase 3 trial. *The Lancet*.

[11] Kandzari DE, Mauri L, Koolen JJ, Massaro JM, Doros G, Garcia-Garcia HM (2017). Ultrathin, bioresorbable polymer sirolimus-eluting stents versus thin, durable polymer everolimus-eluting stents in patients undergoing coronary revascularisation (BIOFLOW V): a randomised trial. *The Lancet*.

[12] Silber S, Serruys PW, Leon MB, Meredith IT, Windecker S, Neumann F (2013). Clinical Outcome of Patients with and without Diabetes Mellitus after Percutaneous Coronary Intervention with the Resolute Zotarolimus-Eluting Stent: 2-year results from the prospectively pooled analysis of the international global RESOLUTE program. *JACC: Cardiovascular Interventions*.

[13] Moretti C, Lolli V, Perona G, Vignolini M, Cabiale K, Falzone M (2012). Cre8™ coronary stent: preclinical in vivo assessment of a new generation polymer-free DES with Amphilimus™ formulation. *EuroIntervention*.

[14] Romaguera R, Gómez-Hospital JA, Gomez-Lara J, Brugaletta S, Pinar E, Jiménez-Quevedo P (2016). A Randomized Comparison of Reservoir-Based Polymer-Free Amphilimus-Eluting Stents Versus Everolimus-Eluting Stents With Durable Polymer in Patients With Diabetes Mellitus: The RESERVOIR Clinical Trial. *JACC: Cardiovascular Interventions*.

